# Intrathecal IGF2 siRNA injection provides long-lasting anti-allodynic effect in a spared nerve injury rat model of neuropathic pain

**DOI:** 10.1371/journal.pone.0260887

**Published:** 2021-12-02

**Authors:** Wei-Hung Chan, Nian-Cih Huang, Yi-Wen Lin, Feng-Yen Lin, Chien-Sung Tsai, Chun-Chang Yeh

**Affiliations:** 1 Department of Anesthesiology, National Defense Medical Center, Taipei, Taiwan; 2 Department of Anesthesiology, Tri-Service General Hospital, Taipei, Taiwan; 3 Institute of Oral Biology, National Yang-Ming Chiao-Tung University, Hsinchu, Taiwan; 4 Department of Internal Medicine and Taipei Heart Institute, Taipei Medical University, Taipei, Taiwan; 5 Division of Cardiology, Taipei Medical University Hospital, Taipei, Taiwan; 6 Division of Cardiovascular Surgery, Tri-Service General Hospital, National Defense Medical Center, Taipei, Taiwan; 7 Department and Graduate Institute of Pharmacology, National Defense Medical Center, Taipei, Taiwan; Max Delbruck Centrum fur Molekulare Medizin Berlin Buch, GERMANY

## Abstract

Previous studies have shown an increase of insulin-like growth factor-2 (IGF2) in animal models of neuropathic pain. We aimed to examine the hypothesis that reducing the expression of IGF2 using intrathecal IGF2 small-interfering RNA (siRNA) would attenuate the development of neuropathic pain in rats after spared nerve injury (SNI). Male Wistar rats were divided into three groups: sham-operated group, in which surgery was performed to cut the muscles without injuring the nerves; SNI group, in which SNI surgery was performed to sever the nerves; and SNI + siRNA IGF2 group, in which SNI surgery was performed, and IGF2-siRNA was administered intrathecally 1 day after SNI. The rats were assessed for mechanical allodynia and cold allodynia 1 day before surgery (baseline), and at 2, 4, 6, 8, and 10 days after siRNA treatment. The rat spinal cord was collected for quantitative polymerase chain reaction and western blot analysis. Compared with the SNI group, rats that received IGF2 siRNA showed a significantly increased SNI-induced paw-withdrawal threshold to metal filament stimulation from Day 4 to Day 10 after SNI surgery. IGF2 siRNA significantly decreased the response duration from the acetone test from Day 2 to Day 10 following SNI surgery. SNI increased IGF2 mRNA expression on Day 2 and increased IGF2 protein expression on Day 8 and Day 10 in the spinal cord of the SNI rats. However, the above-mentioned effects of IGF2 mRNA and protein expression were significantly inhibited in the SNI + IGF2 siRNA group. We demonstrated that intrathecal administration of IGF2 siRNA provided significant inhibition of SNI-induced neuropathic pain via inhibition of IGF2 expression in the spinal cord. The analgesic effect lasted for 10 days. Further exploration of intrathecal IGF2 siRNA administration as a potential therapeutic strategy for neuropathic pain is warranted.

## Introduction

Neuropathic pain is a major public health concern that is defined by the International Association for the Study of Pain (IASP) as ‘pain caused by a lesion or disease of the somatosensory nervous system’ [1]. It is a clinical description which requires a demonstrable lesion or a disease that satisfies established neurological diagnostic criteria [2]. The regulation of neuropathic pain is multifactorial, including genetic [3], epigenetic [4], and environmental [5] factors. Following damage to peripheral nerves, the nociceptive signals, neurotransmitters, and cytokines depolarize the postsynaptic neurons and activate the glial cells of the spinal cord, which causes neuroinflammation. Neuroinflammation involves neural-immune interactions that activate the immune cells, glial cells, and neurons, and plays a key role in the development of neuropathic pain [6, 7].

In our previous study that investigated the impact of pulsed radiofrequency (PRF) on the modulation of pain-regulatory genes after spared nerve injury (SNI) in rats, we demonstrated that PRF treatment significantly inhibited the development of neuropathic pain and down-regulated insulin-like growth factor-2 (IGF2) expression in the dorsal horn of the rat spinal cord [8], suggesting that inhibiting IGF2 expression in the rat spinal cord might play a role in alleviating neuropathic pain. Recently, gene therapies based on small-interfering RNA (siRNA) silencing of disease-related genes have provided hope as a potential treatment for intractable disorders [9]. The functions of siRNA include gene-silencing process targeting and degrading specific mRNA molecules, resulting in the degradation of mRNA. Moreover, it can regulate multiple important biological processes associated with the onset and progression of inflammation [10]. Previous studies have shown that the use of intrathecally injected siRNA targeting specific genes associated with the inflammatory signaling pathway in the central nervous system, such as P2X3 or TLR4 [10, 11], may attenuate neuropathic pain. In this study, we aimed to test the hypothesis that IGF2 siRNAs alleviate neuropathic pain, by verifying its effects on an SNI rat model.

## Materials and methods

### Study design

**Fig 1:**
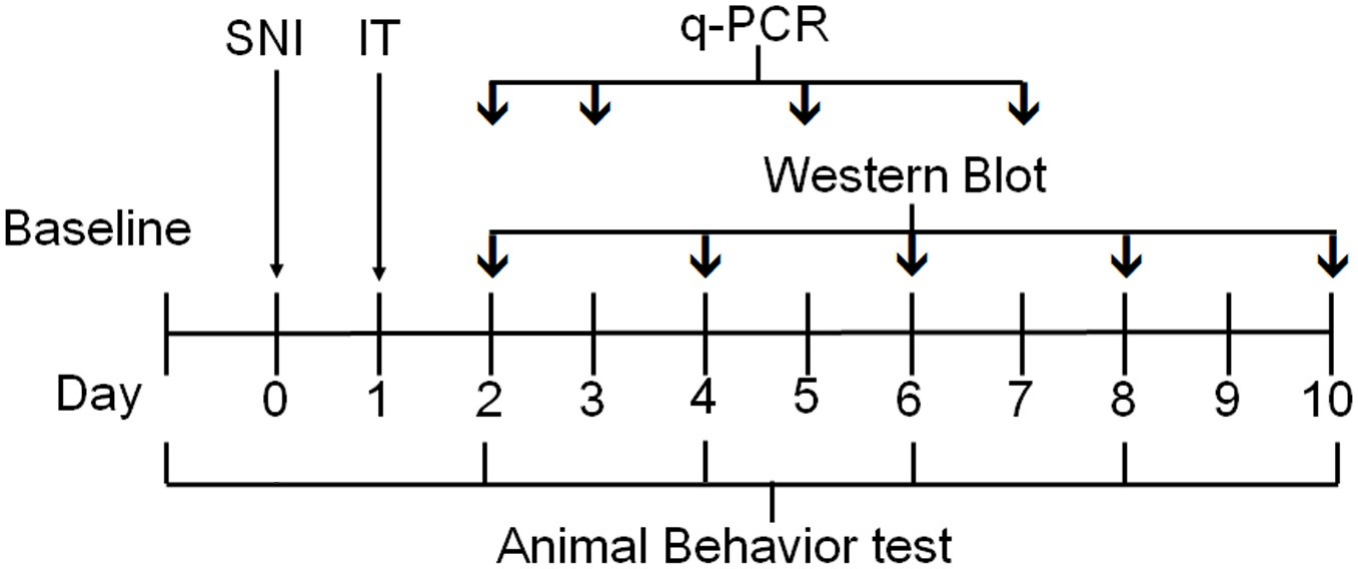
Experiment timeline. Timeline of the experimental protocols, outlining the periods of animal behavior testing, spared nerve injury (SNI) surgery, real-time quantitative polymerase chain reaction (RT-qPCR), and western blot. IT, intrathecal.

Male Wistar rats were divided into three groups as follows: (1) sham-operated group: surgery was performed to cut the muscles without injuring the nerves; (2) SNI group: SNI surgery was performed to sever the nerves; (3) SNI + siRNA IGF2 group: SNI surgery was performed, and IGF2-siRNA was administered by intrathecal injection once a day after SNI. The animals were assessed for mechanical allodynia using a dynamic plantar aesthesiometer (DPA), and for cold allodynia using the acetone spray test 1 day before surgery (baseline), and at 2, 4, 6, 8, and 10 days after siRNA treatment. The rat spinal cord tissue was collected for quantitative polymerase chain reaction (qPCR) on Days 2, 3, 5, and 7 and western blot analysis on Days 2, 4, 6, 8, and 10. The experiment timeline is shown in Fig 1.

### Animals

All experimental procedures were conducted in accordance with the Guide for the Care and Use of Laboratory Animals, published by the National Institutes of Health (Bethesda, ML, USA) and after approval of the Animal Care and Use Committee of the National Defense Medical Center (Taipei, Taiwan, Republic of China, approval number: IACUC-19-055). Male Wistar rats (BioLASCO, Taipei, Taiwan) weighing 200–250 g were housed in groups of three (but individually after surgery) under standard conditions, with soft bedding in a 12 h light/dark cycle with food and water available ad libitum for 7 days, for acclimatization before the experiment. All efforts were made to minimize the number of animals used and their suffering [12]. The rats were randomly divided into three groups: the SNI group, SNI + siRNA IGF2 group, and the sham-operated group.

### SNI model of neuropathic pain

Neuropathic pain was induced using the SNI model, as described by Decosterd and Woolf [13]. Each rat was anesthetized with 1.5% isoflurane (Halocarbon, River Edge, NJ, USA) and 1 L/min oxygen in a warmed induction chamber over approximately 5 min. The anesthesia depth was confirmed once the rat lost its righting reflex and the breathing pattern became deeper and slower. The rat was transferred to a non-rebreathing circuit with nose cone and the isoflurane percentage was adjusted to the level required to maintain a stable surgical plane of anesthesia. The rat was placed on a heating pad and its core temperature was measured to reduce heat loss. The fur on the lateral surface of the left thigh was shaved using a blade. After aseptic skin preparation by topical application of a povidone-iodine prep pad, the skin was incised and dissection through the biceps femoris muscle was performed to expose the left sciatic nerve and its three terminal branches: the sural, common peroneal, and tibial nerves at the mid-thigh level. The common peroneal and the tibial nerves were tightly ligated with a 4–0 silk suture and axotomized distal to the ligation; a 2–4 mm piece of each distal nerve stump was removed, while the sural nerve was left intact. The muscle and skin were closed in two layers with 4–0 silk sutures. The Sham operation followed the same protocol but did not involve nerve injury. The duration of the entire procedure from induction to recovery was about 15–20 minutes. After the surgery, the rats recovered fully from anesthesia before being returned to their home cage.

### Behavioral testing

Mechanical allodynia was examined using a DPA (Ugo Basile, Comerio, Italy), which is an automated version of the von Frey filament that does not produce tissue damage [14, 15] but produces non-noxious tactile stimuli [16]. Each rat was kept in a transparent plastic cage (25 cm × 10 cm × 14 cm) with a wire mesh grid and acclimated to the cage for 15 min before each test procedure. A paw-withdrawal response was elicited by applying an increasing force using a blunt-end metal filament (diameter, 0.5 mm) focused on the region of the sural nerve at the plantar surface of the left ipsilateral hind paw. The force was linearly increased (at a rate of 2.5 g/s to a maximum of 50 g, then held for an additional 10 s at 50 g) until a withdrawal reflex was evoked. Mechanical thresholds were determined by paw withdrawal to metal filament stimulation of the glabrous surface of the paw. Only a quick flick was considered as a response, and movements related to locomotor activity were ignored. (each application was repeated for three times at 1-min intervals to determine the threshold) [17].

Cold allodynia was determined by measuring the duration of hind paw withdrawals in response to acetone application. The rats were placed in a transparent plastic cage on top of a wire mesh floor and were acclimatized to the environment for 15 min before performing the test. Cold allodynia was assessed by vaporizing acetone (100 μL) onto the lateral plantar surface (surally innervated area) of the ipsilateral hind paw with an Eppendorf multi-stepper pipette. The duration of behavior (shaking, flinching, biting, or licking) that ensued over a 1-min period was recorded [18, 19]. Each trial was repeated three times at a minimal 5-min interval. A minimal value of 0.5 s was allocated for a fast or brisk reaction; a value of 0 s was allocated for no reaction at all [17].

### SiRNA knockdown of IGF2 expression and intrathecal treatment

The siRNA against IGF2 was synthesized and purchased from MD Bio, Inc. (Taipei, Taiwan). The following siRNA sequences were used: IGF2 forward, 5’- CUGAUCGUGUUACCACCCAAAtt-3’. SiRNA was administered to the rats in the SNI + siRNA group through intrathecal injections once a day after SNI. Intrathecal injections were administered as previously described [20] with some modifications. After shaving and sterilizing the back around L4-5, the rats were anesthetized with 1.5% isoflurane, and intrathecal injections were made by lumbar puncture into the L4-5 intervertebral space using a 21-gauge scalp vein set (Nipro Co.) fitted to a Hamilton 25-μL syringe. Before injection, the scalp vein set was filled with 10 μL 2% lidocaine first, followed by 10 μL of either siRNA or saline, and a final 5 μL 2% lidocaine by withdrawing the syringe from the needle. A correct injection was confirmed when a 10–15 minutes bilateral hindlimb motor deficit developed within 1 min after the injection. The optimal administration dosages of siRNA were selected according to a previous study [20].

### Harvesting of the dorsal horn of the spinal cord

All rats were sacrificed using euthanasia methods immediately after the behavioral tests on different days of completion. The rats were anesthetized with 4% isoflurane in an induction chamber. After the confirmation of unconsciousness and respiratory cessation for at least 3 minutes, exsanguination of the rats via cardiac venipuncture was performed. The ipsilateral dorsal quadrant of the lumbar spinal cord enlargement was dissected and removed. The dorsal horn was collected in microcentrifuge tubes, snap-frozen in liquid nitrogen, and stored at −80°C until further use [17].

### Real-time quantitative PCR (RT-qPCR)

On Days 2, 3, 5, and 7, the total RNA from the spinal cord samples (L4-L6) was extracted using Trizol reagent (Invitrogen, Carlsbad, CA, USA). Two micrograms of the total RNA were reverse transcribed according to the manufacturer’s protocol (Invitrogen). qPCR analysis was performed in the QuantStudio 3 Real-Time PCR System (Thermo Fisher Scientific Inc., Rockford, IL, USA) by Fast SYBR^™^ Green Master Mix (Invitrogen, Carlsbad, CA). The following primers were used: IGF2 forward, 5’-ATTCGACACCTGGAGACAGTC-3’; IGF2 reverse, 5’- GTTGCTGGACATCTCCGAAGAG-3’ The PCR amplifications were performed at 95°C for 30 s, followed by 35 cycles of thermal cycling at 95°C for 20 s and 60°C for 20 s. Glyceraldehyde 3-phosphate dehydrogenase was used as an endogenous control to normalize the differences for mRNA expression. Melt curves were performed on completion of the cycles to ensure that nonspecific products were absent.

### Western blot

On Days 2, 4, 6, 8, and 10, the rats were sacrificed, and the total proteins were extracted by homogenizing the spinal cord samples (L4–6) in a radioimmunoprecipitation assay buffer containing 1 mM phenylmethyl sulfonyl fluoride, and 1X protease inhibitor cocktail (Sigma, St. Louis, MO, USA). Protein concentrations were determined using a Bio-Rad Protein Assay Kit (BioRad Inc., CA, USA), with bovine serum albumin used as the standard. For each blot, approximately 35 μg of total protein was fractionated by SDS-PAGE and transferred to a polyvinylidene difluoride membrane. The membranes were probed with rabbit anti-IGF2 (ab9574, Abcam, MA, USA) antibody, stored at 4°C overnight, and washed with phosphate-buffered saline containing 0.1% Tween-20. The membranes were then incubated with horseradish peroxidase-conjugated goat anti-rabbit IgG (Merck Millipore, Darmstadt, Germany) antibody. The proteins were visualized using a chemiluminescence system (Millipore, Bedford, MA, USA) and exposed to a ChemiDoc-It 515 Imaging System (Ultra-Violet Products Ltd., Cambridge, UK). Mouse anti-β-actin antibody (A5441, Sigma-Aldrich, St. Louis, MO, USA) was used as the loading control.

### Statistical analysis

Statistical analyses were carried out using SPSS software (version 14.0, SPSS Inc., Chicago, IL, USA) and Prism Version 8.4.3 (GraphPad, USA). Behavioral test data are presented as means ± standard deviations (SDs). The results of behavioral test were analyzed by two-way repeated measures ANOVA followed by Bonferroni test for intergroup comparisons of normally distributed variants. The Kruskal-Wallis test was applied to non-normally distributed variants. One-way ANOVA test was used to analyze the results of the qPCR and Western blot tests. For all the above tests, statistical significance was set at *P* < 0.05.

## Results

### Intrathecal injection of IGF2 siRNA alleviated mechanical allodynia and cold allodynia in SNI model of rats

**Fig 2:**
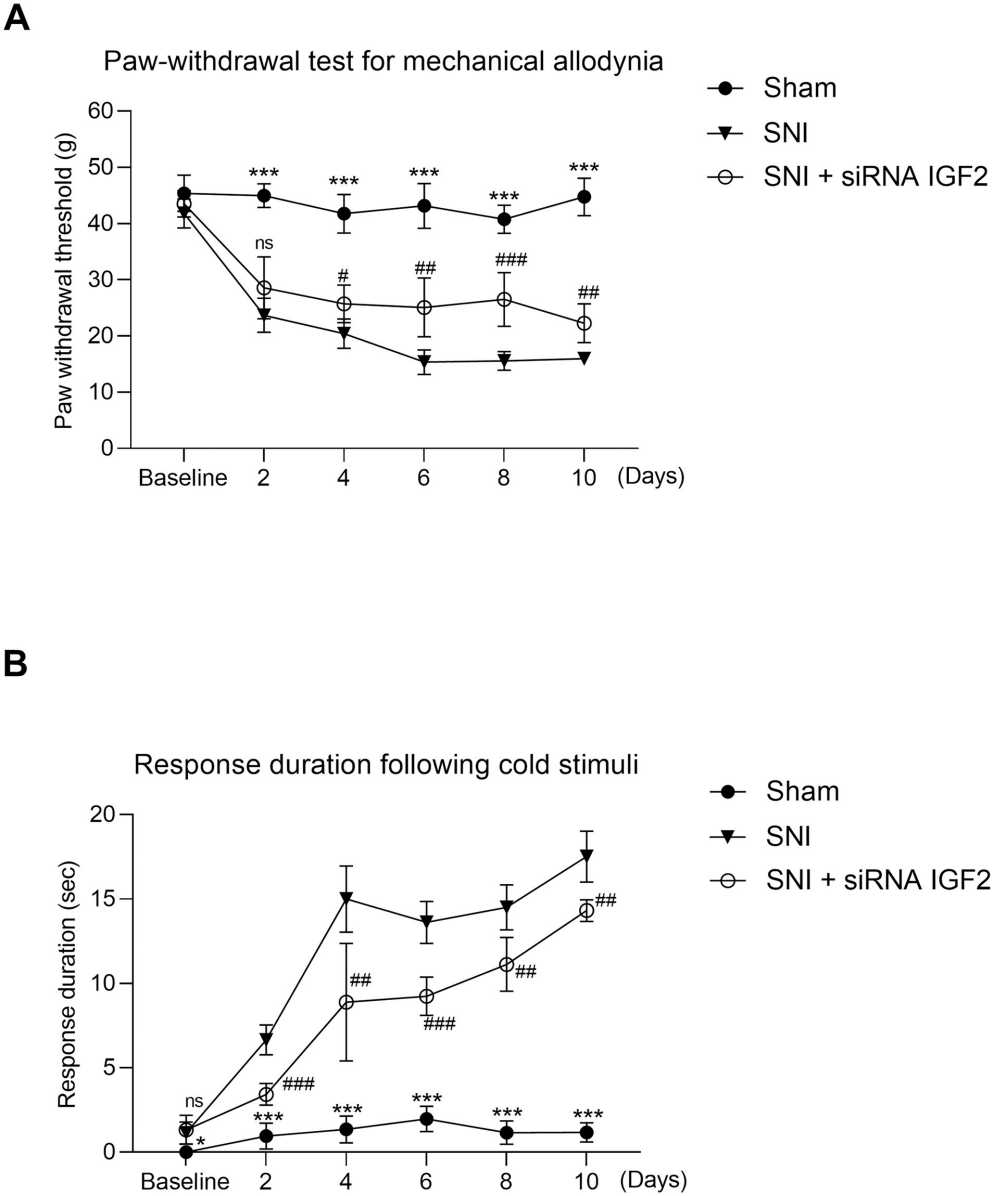
Effect of intrathecal injection of IGF2 siRNA on rat behavior. Rats are randomly divided into three groups (n = 6 per group): sham (normal saline solution) group (●); SNI group (normal saline solution) (▼); SNI + siRNA IGF2 group (○). **A**. Paw-withdrawal test for mechanical allodynia. Mechanical allodynia is examined using a dynamic plantar aesthesiometer in the three groups. SNI versus sham ***P < 0.001; SNI + siRNA IGF2 #*P* < 0.05, ##*P* < 0.01, ###*P* < 0.001. **B**. Response duration following cold stimuli using an acetone spray test in the three groups. SNI versus sham *P <0.05, ***P < 0.001; SNI + siRNA IGF2 #*P* < 0.05, ##*P* < 0.01, ###*P* < 0.001. Each point represents the mean ± SD. The data presented above were examined by two-way repeated measures ANOVA followed by Bonferroni post hoc comparisons. SNI, spared nerve injury; siRNA, small-interfering RNA; IGF2, insulin-like growth factor-2; ns, not significant.

The values of animal behavior are presented in Table 1 and Fig 2. Repeated Measures ANOVA detected significant effects of time (F (5, 11) = 145.639, *p* < 0.001) and group (F = 284.490, *p* < 0.001), and a significant time × group interaction for the paw-withdrawal threshold (PWT) (F = 14.827, *p* < 0.001). Compared with the sham group, the PWT was significantly lower in the SNI group, beginning at Day 2 (45.0 ± 2.1 g versus 23.7 ± 3.0 g, sham group versus SNI group; *p* < 0.001) and lasting 8 days. Compared with the SNI group, the PWT was significantly higher in the SNI + siRNA IGF2 group, beginning at Day 4 (25.76 ± 3.4 g) and lasting for 6 days (D10) (Table 1A, Fig 2A). Repeated Measures ANOVA detected significant effects of time (F (5, 11) = 542.525, *p* < 0.001) and group (F = 895.420, *p* < 0.001), and a significant time × group interaction for the response duration from the acetone test (F = 35.782, *p* < 0.001). Compared with the sham group, the response duration from the acetone test was significantly longer in the SNI group, beginning at Day 2. Compared with the SNI group, the response duration from the acetone test was significantly shorter in the SNI + siRNA IGF2 from D2 to D10 (Table 1B, Fig 2B).

**Table 1:** Behavioral testing.

A. Paw-withdrawal test for mechanical allodynia						
Days after SNI	Paw-withdrawal threshold (g)			Adjusted *P*-value		
	Sham	SNI	SNI + siRNA IGF2	1	2	3
BL	45.4±3.2	41.8±2.6	43.5±2.3	0.112	0.893	0.739
D2	45.0±2.1	23.7±3.0	28.6±5.5	<0.001	0.128	<0.001
D4	41.7±3.4	20.4±2.6	25.7±3.4	<0.001	0.031	<0.001
D6	43.2±4.0	15.3±2.2	25.1±5.2	<0.001	0.002	<0.001
D8	40.8±2.5	15.6±1.6	26.5±4.8	<0.001	<0.001	<0.001
D10	44.8±3.3	16.0±0.7	22.3±3.5	<0.001	0.004	<0.001
B. Response duration following cold stimuli						
Days after SNI	Painful behavior time (sec)			Adjusted *P*-value		
	Sham	SNI	SNI + siRNA IGF2	1	2	3
BL	0	1.2±0.6	1.3±0.9	0.018	1.000	0.006
D2	1.0±0.8	6.7±0.9	3.4±0.6	<0.001	<0.001	<0.001
D4	1.4±0.8	15.0±2.0	8.9±3.5	<0.001	0.001	<0.01
D6	2.0±0.7	13.6±1.2	9.2±1.1	<0.001	<0.001	<0.001
D8	1.2±0.7	14.5±1.3	11.1±1.6	<0.001	0.001	<0.001
D10	1.2±0.6	17.5±1.5	14.3±0.7	<0.001	0.004	<0.001

SNI, spared nerve injury; siRNA, small-interfering RNA; IGF2, insulin-like growth factor-2; BL, baseline; D, day; Values are mean ± standard deviation; n = 6; adjusted *P*-value 1: sham group compared with SNI group; adjusted *P*-value 2: SNI + siRNA IGF2 group compared with SNI group; adjusted *P*-value 3: SNI + siRNA IGF2 group compared with sham group; The data presented above were examined by ANOVA followed by Bonferroni post hoc comparisons. P<0.05: statistically significant.

### SNI increased IGF2 expression in the spinal cord of SNI rats

**Fig 3:**
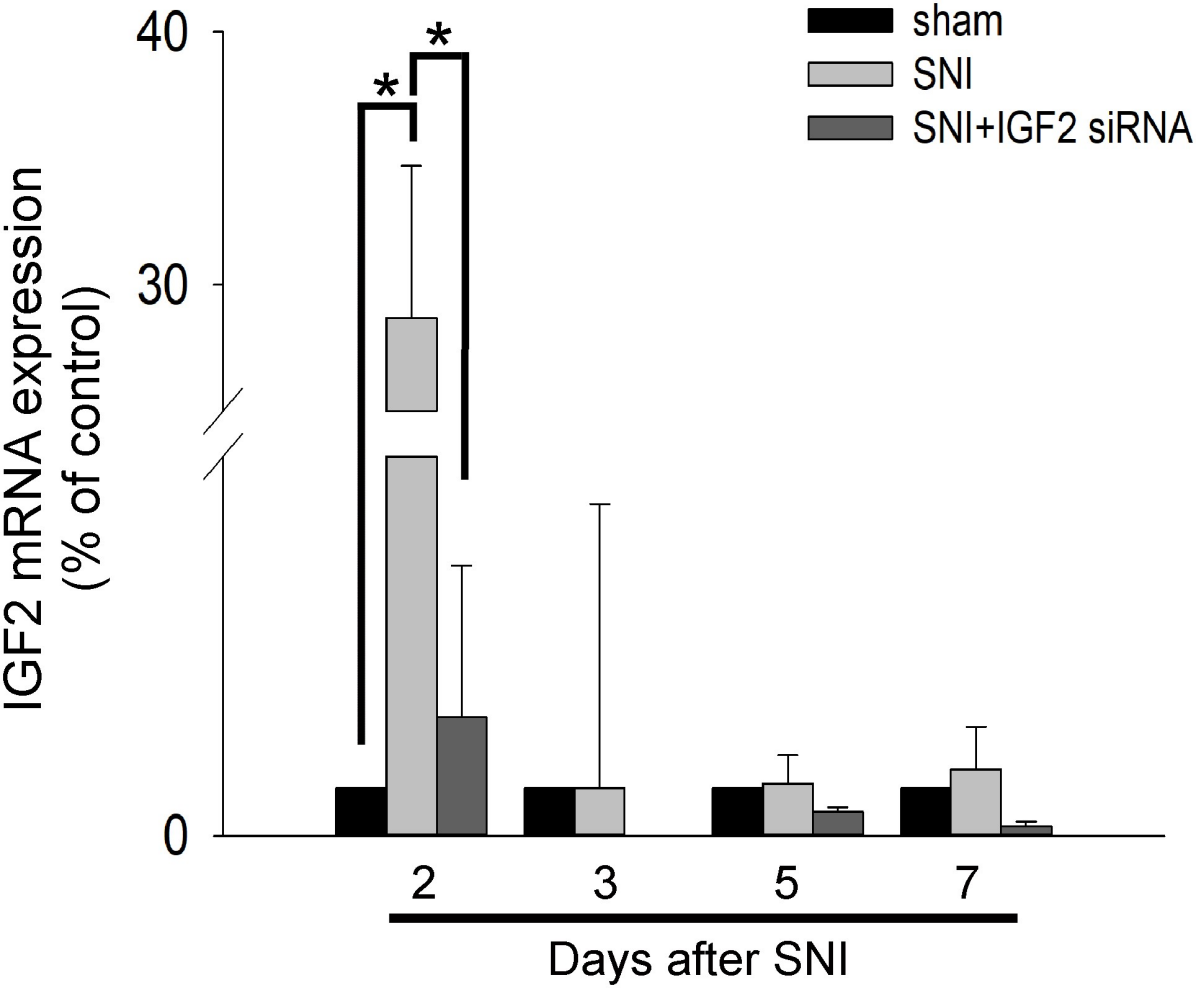
Intrathecal injection of IGF2 siRNA decreases IGF2 mRNA expression in the spinal cord. On Days 2, 3, 5, and 7, the rats are sacrificed, and the total RNA of the spinal cord is extracted by homogenizing spinal cord samples (L4–6). Data are presented as mean ± standard deviation (n = 3 per group). Black bar, sham group; gray bar, SNI group; dark bar, SNI + siRNA IGF2 group. SNI, spared nerve injury; siRNA, small-interfering RNA; IGF2, insulin-like growth factor-2.

To explore the role of IGF2 in neuropathic pain, we evaluated IGF2 mRNA expression in the spinal cord from Day 2 to Day 7 after SNI surgery using RT-qPCR. The results showed an increase in the expression of IGF2 mRNA after SNI surgery, compared with the sham group. The increase in expression was only significant on Day 2 and was significantly inhibited after intrathecal IGF2 siRNA administration (Table 2 and Fig 3).

**Table 2:** IGF2 mRNA expression by RT-qPCR.

Days after SNI	IGF2 mRNA expression (fold of sham)			P-value	
	Sham	SNI	SNI + siRNA IGF2	1	2
D2	1	28.7±6.0	2.5±3.2	<0.001	<0.001
D3	1	1.0±1.0	0	0.988	0.421
D5	1	1.1±0.6	0.5±0.1	1.0	0.134
D7	1	1.4±0.9	0.2±0.1	0.562	0.293

Values are mean ± standard deviation; n = 4; P-value 1: sham group compared with SNI group; P-value 2: SNI + siRNA IGF2 group compared with SNI group. P < 0.05: statistically significant. SNI, spared nerve injury; siRNA, small-interfering RNA; IGF2, insulin-like growth factor-2; BL, baseline; D, day; RT-qPCR, real-time quantitative polymerase chain reaction.

**Fig 4:**
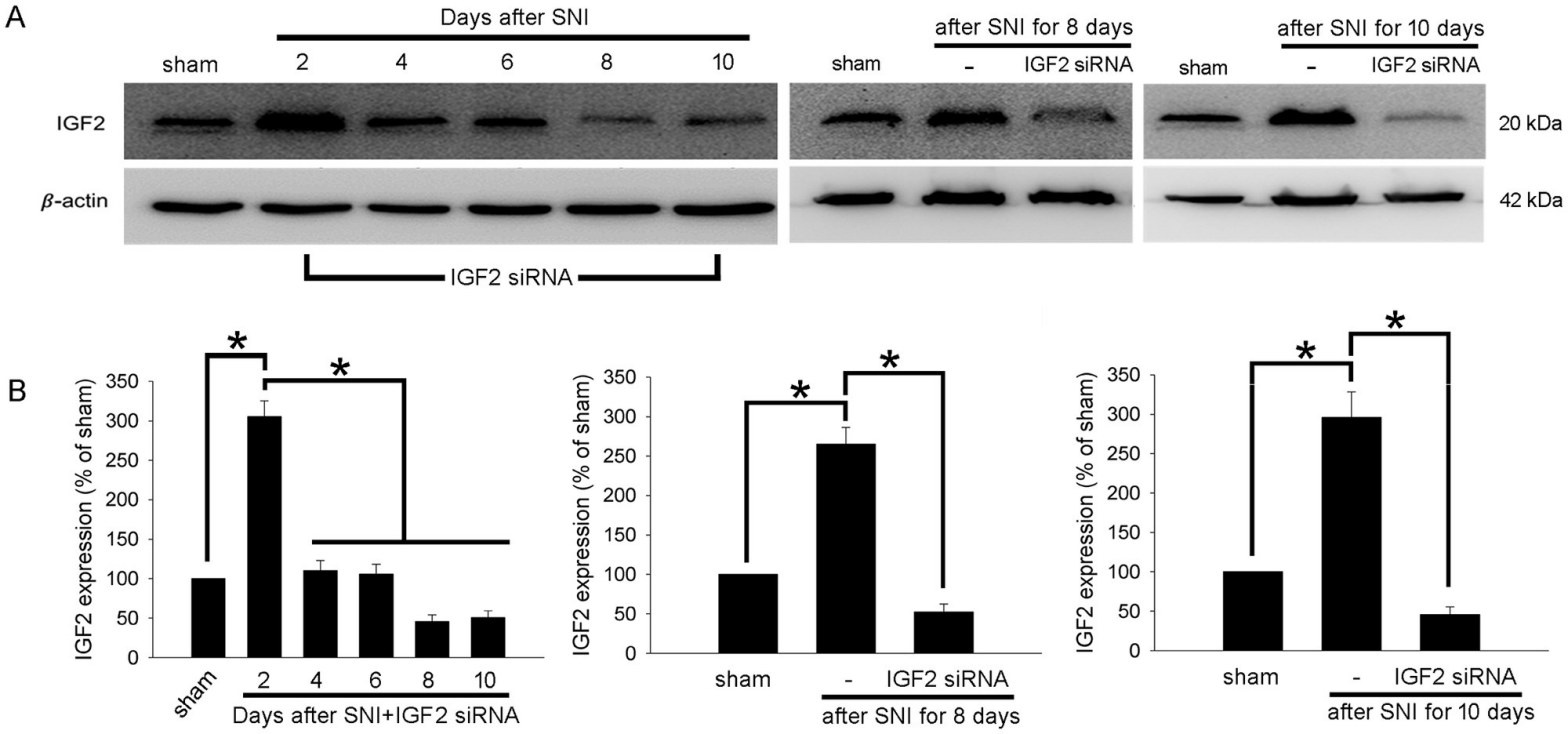
Intrathecal injection of IGF2 siRNA decreases IGF2 protein expression in the spinal cord. Western blotting analysis for IGF2 in protein extracts from spinal cord tissue in different groups and timepoints. (A) Left, comparison of IGF2 protein expression levels in the spinal cord dorsal horn in the sham group and in the SNI + IGF2 siRNA group (on Days 2, 4, 6, 8, and 10). Middle, comparison of IGF2 protein expression levels in the spinal cord dorsal horn in the sham group, SNI group and the SNI + IGF2 siRNA group (after SNI for 8 days). Right, comparison of IGF2 protein expression levels in the spinal cord dorsal horn in the sham group, SNI group and the SNI + IGF2 siRNA group (after SNI for 10 days). Representative blots are shown. (B) Quantitative analysis of IGF2 expression shown in (A). The IGF2/β-actin ratio in the sham group is set to 100%. Data are presented as mean ± standard deviation (n = 3); *P < 0.05 corresponds to significant difference. siRNA, small-interfering RNA; IGF2, insulin-like growth factor-2.

To investigate the effect of IGF2 gene knockdown by IGF2 siRNA on the protein level, rats of the SNI + siRNA IGF2 group were sacrificed after behavior testing on Days 2, 4, 6, 8, and 10, and spinal cord samples were obtained for western blot and compared with the sham group (n = 3). The results showed that compared with the sham group, the IGF2 protein expression was still significantly increased 2 days after the SNI surgery in the SNI + IGF2 siRNA group (Fig 4A and 4B, left). However, compared with Day 2, the IGF2 protein expression decreased significantly from Day 4 to Day 10 in the SNI + IGF2 siRNA group. On Day 8 and Day 10, the IGF2 expression level in the SNI + IGF2 siRNA group was even lower than the level in the sham group (Fig 4A and 4B, left). Further, protein expression analyses between groups on Day 8 and Day 10 showed that IGF2 increased significantly in the SNI group compared with the sham group. However, IGF2 expression was significantly inhibited in the SNI + IGF2 siRNA group compared with the SNI group (Fig 4A and 4B, middle and right).

## Discussion

IGF2 is a peptide hormone that belongs to the IGF family of proteins [21, 22]. Both IGF1 and IGF2 regulate somatic growth and cell proliferation by activating receptor tyrosine kinases, which are involved in early body development [21, 23]. Moreover, IGF2 is also known to be expressed in glial cells and is involved in neural plasticity [1] and neuroinflammation [8, 22]. An atherosclerosis study by Qiao *et al*. has shown that IGF2 inhibition reduces lipid accumulation and inflammatory responses by inhibiting NF-κB expression in macrophages [24]. A study by Suh *et al*. indicated that human microglial IGF2 mRNA and protein were upregulated by proinflammatory mediators such as lipopolysaccharide (LPS) [22]. Additionally, our previous study demonstrated that co-localized phosphorylated ERK1/2 and IGF2 in the dorsal horn regions of neuropathic pain rat models and exogenous IGF2-treated glioblastoma cells induced ERK1/2 phosphorylation [8], which has been implicated in various conditions, including pain and neuroinflammation [25]. Therefore, we speculated that IGF2 has a close relationship with neuropathic pain.

In this study, we investigated the effects of intrathecal IGF2 siRNA injection [26–28] on neuropathic pain behavior and knockdown efficiency in an SNI rat model. We observed that SNI produced lasting mechanical allodynia and cold allodynia while intrathecal IGF2 siRNA administration significantly alleviated neuropathic pain behavior by inhibiting IGF2 expression in the rat spinal cord. Our previous results have suggested that pulsed radiofrequency (PRF) treatment immediately after SNI in a rat model significantly inhibited the development of neuropathic pain with a lasting effect, most likely through IGF2 downregulation in the spinal cord [8]. However, the causality of spinal cord IGF2 expression and neuropathic pain was unclear. Our results further confirmed that IGF2 in the spinal cord contributes to neuropathic pain.

Mechanical allodynia and cold allodynia both develop following peripheral nerve injury and are major concerns in neuropathic pain [29, 30]. In our findings, the PWT was significantly lower in the SNI rats beginning at Day 2, compared with the sham group, suggesting increased sensitivity to mechanical stimuli and correlation with mechanical allodynia. Development of cold allodynia after SNI was significantly immediate (Table 1B, Fig 2B). The relatively quick development of cold allodynia, compared with mechanical allodynia, was consistent with the findings of a previous study about mechanistic differences in neuropathic pain [30]. Cobos *et al*. observed that TrpV1-lineage neurons participate in cold allodynia and immune system (e.g., T cell) activation, thus contributing to mechanical allodynia. They also demonstrated the early onset of cold allodynia and late onset of mechanical allodynia. IGF2 participates in central [31] and peripheral [32] nervous system development and may intervene in the control of T cell differentiation [33]. Therefore, it is reasonable that modulating IGF2 expression in the spinal cord may alter these two neuropathic pain modalities. In our study, IGF2 siRNA showed a significant anti-allodynia effect 3 days after intrathecal administration (Day 4) in the PWT test, and 1 day after intrathecal administration (Day 2) in the acetone test, and lasted over 6 days (Day 10).

IGF2 mRNA expression significantly increased on Day 2 after the SNI surgery while the expression returned to baseline level 3 days after SNI (Table 2 and Fig 3). However, IGF2 protein expression still increased significantly 10 days after SNI compared with the sham group and SNI + IGF2 siRNA group (Fig 4A and 4B, right). The results supported the significant difference of neuropathic pain behavior between the SNI group and the other two groups. On the other hand, the expression of IGF2 mRNA was significantly inhibited after intrathecal IGF2 siRNA administration (Fig 3). However, the IGF2 protein expression on Day 2 in the SNI + siRNA IGF2 group was still significantly increased (compared with the sham group) and decreased gradually (Fig 4A and 4B, left). This inconsistency in the results may be related to the different half-lives of the IGF2 mRNA and protein, post-transcriptional modification, and protein synthesis or folding. The function of IGF2 protein was also supported by the behavioral findings of the rats. However, although the IGF2 protein expression in the SNI + IGF2 siRNA group decreased to the same level as in the sham group on Day 8 and 10 (Fig 4A and 4B, left), the behavior associated with mechanical and cold allodynia did not return to baseline level (Table 1, Fig 2). These results suggest that there may be several other mechanisms contributing to neuropathic pain.

Management of neuropathic pain includes pharmacotherapy, such as gabapentinoids, tricyclic antidepressants, and selective serotonin–norepinephrine reuptake inhibitors [34]. Several preclinical studies have focused on inhibiting the inflammatory pathway and assessed medications with anti-inflammatory effects [2]. For symptoms poorly controlled by oral drug administration, invasive treatments such as intrathecal morphine pump [35], spinal cord stimulation [36], and PRF [37] have also been applied in clinical practice with long-term therapeutic effects. However, these procedures may be skill-dependent, time-consuming, and relatively inconvenient for the patient. Recently, the potential of molecular targeting therapy, such as siRNA prescription, for pain management has been established [37, 38]. However, efficient delivery of siRNAs to the central nervous system remains a challenge, since siRNAs do not readily cross the brain–blood barrier and are rapidly degraded *in vivo* [39]. Besides the functions of IGF2 as mentioned above, a previous study demonstrated that systemic injections of IGF2 in mice significantly increase short- and long-term memory [40]. Therefore, systemic administration of an IGF2 inhibitor might result in unwanted side effects. Studies have shown that intrathecal siRNA administration is a new approach to target neuropathic pain [41] by downregulating gene expression in the central nervous system, and hence, relieving pain [10, 26–28, 42]. A study by William *et al*. reported that intrathecal administration of P2X_3_ siRNA inhibited P2X_3_ mRNA expression in dorsal root ganglia, and hence, suppressed mechanical hyperalgesia in rats [10]. A study by Yu *et al*. revealed that suppression of TLR4 by intrathecal siRNA injection attenuated chronic constriction injury-induced mechanical allodynia and thermal hyperalgesia through inhibiting NF-κB activation and proinflammatory cytokine production [11]. Both P2X_3_ and TLR4 play a role in inflammatory pain signaling in the spinal cord [43, 44]. There are certain limitations to our study. First, our previous study showed that IGF2 is present in microglial and neuronal cells in the spinal horn and plays a role in ERK1/2 phosphorylation [8]. However, the upstream pathway that modulates IGF2 expression or the downstream pathway that connects IGF2 and neuroinflammation in neuropathic pain was not investigated. For example, whether ERK1/2 phosphorylation in the spinal cord is inhibited by the attenuation of IGF2 expression using siRNA in SNI rats needs to be further studied. Second, T cell immune responses potentially contribute to neuropathic pain [45]. Since IGF2 may participate in the control of T cell differentiation [33], the role of IGF2 in the interaction between neurons, microglia, and T cells should be further investigated.

## Conclusions

To the best of our knowledge, this is a pioneering study that demonstrated that intrathecal IGF2 siRNA injection effectively inhibited IGF2 protein and mRNA expression in the spinal cord and attenuated allodynia in an SNI rat model, as observed in our behavioral findings. Targeting IGF2 by intrathecal siRNA administration may be an innovative and applicable therapeutic strategy with an acceptable duration of therapeutic effect for neuropathic pain management. In the future, further investigations that include an analysis of the side effects of intrathecal IGF2 injection and that of cost-effectiveness in other animal models or in humans should be explored.

## Supporting information

S1 Raw images (PDF)
