# Supplementary material for: Intrathecal IGF2 siRNA injection provides long-lasting anti-allodynic effect in a spared nerve injury rat model of neuropathic pain
Source: PLoS One. 2021 Dec 2;16(12):e0260887. doi: 10.1371/journal.pone.0260887 (PMC8638935; doi:10.1371/journal.pone.0260887)

Figure 4 left-upper

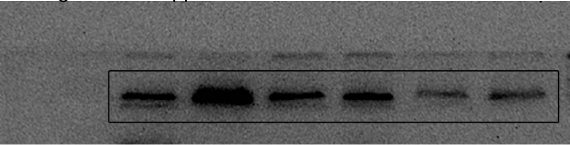

Figure 4 left-lower

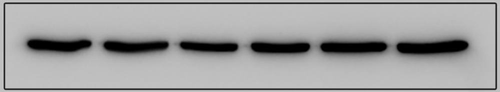

Figure 4 middle-upper

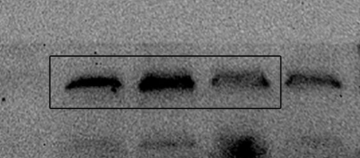

Figure 4 middle-lower

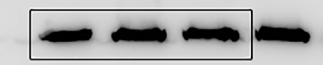

Figure 4 right-upper

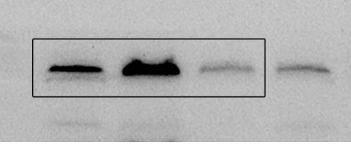

Figure 4 right-lower

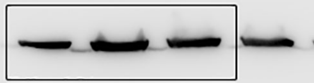

Supplement: S1 Raw images — (PDF) [file pone.0260887.s001.pdf]
